# Is fluoroscopy‐guided percutaneous bone biopsy of diabetic foot with suspected osteomyelitis worthwhile? A retrospective study

**DOI:** 10.1111/1753-0407.13377

**Published:** 2023-03-10

**Authors:** Hassan Al‐Balas, Zeyad A. Metwalli, Aaditya Nagaraj, David M. Sada

**Affiliations:** ^1^ Jordan University of Science and Technology Irbid Jordan; ^2^ University of Texas M.D. Anderson Cancer Center Houston Texas USA; ^3^ Baylor College of Medicine Houston Texas USA; ^4^ Michael E. DeBakey VA Medical Center Houston Texas USA

**Keywords:** diabetic foot, diabetic foot ulcer, foot osteomyelitis, percutaneous bone biopsy, 糖尿病足, 足溃疡, 骨髓炎, 经皮骨活检

## Abstract

**Background:**

Diabetic foot infection, particularly osteomyelitis, is a major risk factor of amputation in persons with diabetes. Bone biopsy with microbial examination is considered the gold standard of diagnosis of osteomyelitis, providing information about the offending pathogens as well as their antibiotics susceptibility. This allows targeting of these pathogens with narrow spectrum antibiotics, potentially reducing emergence of antimicrobial resistance. Percutaneous fluoroscopy guided bone biopsy allows accurate and safe targeting of the affected bone.

**Methods:**

In a single tertiary medical institution and over 9 year period, we performed 170 percutaneous bone biopsies. We retrosepctively reviewed the medical record of these patients including patients' demographics, imaging and biopsy microbiology and pathollogic results.

**Results:**

Microbiological cultures of 80 samples (47.1%) were positive with 53.8% of the positive culture showed monomicrobial growth and the remaining were polymicrobial. Of the positive bone samples 71.3% grew Gram‐positive bacteria. *Staphylococcus aureus* was the most frequently isolated pathogen from positive bone cultures with almost one third showing methicillin resistence. *Enterococcus* species were the most frequently isolated pathogens from polymicrobial samples. *Enterobacteriaceae* species were the most common Gram‐negative pathogens and were more common in polymicrobial samples.

**Conclusions:**

Percutaneous image‐guided bone biopsy is a low‐risk, minimally invasive procedure that can provide valuable information about microbial pathogens and therefore enable targeting these pathogens with narrow spectrum antibiotics.

## Abstract 2

**Highlights**

- Bone biopsy of the infected diabetic foot is the gold standard for diagnosis of osteomyelitis with *Staphylococcus aureus* being the most common offending pathogen.
- Percutaneous fluoroscopy‐guided bone biopsy is a potentially useful, minimally invasive procedure to confirm the clinical diagnosis of osteomyelitis and provide valuable microbiological information.
- Bone biopsy in the diabetic foot can help guide antibiotic treatment.

## 摘要

**背景:**糖尿病足感染尤其是骨髓炎是糖尿病患者截肢的主要危险因素。骨活检联合微生物检查是骨髓炎诊断的金标准, 可提供致病病原体及其对抗生素的敏感性信息。这使我们可以用窄谱抗生素靶向这些病原体, 减少抗菌素耐药性的出现。经皮透视引导下的骨活检可安全、准确地定位患骨。

**方法:**在一家三级医疗机构, 我们9年内进行了170例经皮骨活检。我们回顾了这些患者的医疗记录, 包括患者的人口统计数据、影像以及活检微生物学和病理学结果。

结果:在阳性骨样本中, 71.3%为革兰氏阳性细菌。金黄色葡萄球菌是最常见的分离病原体来自阳性骨培养, 几乎三分之一显示甲氧西林耐药。肠球菌是最常见的病原菌多种微生物样本。肠杆菌科细菌是最常见的革兰氏阴性病原体在多种微生物样本中更常见。

**结论:**经皮影像引导骨活检是一种低风险的微创程序, 可以提供关于致病微生物的有价值信息, 用窄谱抗生素靶向这些病原体。

## 1 BACKGROUND

Diabetic foot complications are a major cause of morbidity in persons with diabetes. Up to 25% of these persons develop foot ulcers during their lifetime.^1^ Multiple factors contribute to development of foot ulcers in persons with diabetes including trauma, neuropathy with loss of protective sensation, and peripheral vasculopathy. Infection is the most common complication of diabetic ulcer and will affect 60% of ulcers. Diabetic foot ulcer infection is the leading cause of amputation in this population.^2^, ^3^, ^4^

Osteomyelitis complicating diabetic foot ulcer is typically a result of direct bacterial extension into the exposed bone. Several clinical and imaging techniques and modalities have been proposed to diagnose osteomyelitis with variable accuracy including ability to probe to bone, radiography, magnetic resonance imaging (MRI) and nuclear imaging.^5^, ^6^, ^7^, ^8^, ^9^, ^10^ Bone biopsy with histopathological and microbiological examination remains the gold standard to diagnose osteomyelitis. Fluoroscopy‐guided percutaneous bone biopsy is a simple minimally invasive technique, which can be used to confirm the diagnosis histologically and provide details regarding the offending pathogens. This can help guide antibiotic therapy specifically targeting these microorganisms.

The aim of this study is to report the yield of percutaneous bone biopsy in persons with diabetes with clinically suspected osteomyelitis involving the foot and the spectrum of offending pathogens recovered from these biopsies.

## 2 METHODS

Institutional review board approval was obtained for this retrospective study. The study was performed at a single tertiary Veterans Affairs hospital. Between January 2010 and December 2019, 170 fluoroscopy‐guided biopsies of bones of the foot in 170 persons with diabetic foot ulcers with clinically suspected osteomyelitis were performed in the interventional radiology department. We retrospectively reviewed patient electronic medical records using the Veterans Affairs electronic medical record system, Computerized Patient Record System, and collected demographic information including patient age and gender and biopsy‐related details including site of the biopsy, history of previous imaging, bone specimen histopathology, and microbiological results. Association between pathological diagnosis of osteomyelitis and positive bone cultures and any association of prior use of antibiotics and duration antibiotics were withheld prior to bone biopsy on the positivity of bone culture was assessed using Pearson chi‐square calculation. All statistical analysis was performed using SPSS (IBM SPSS Statistics for Windows, Version 27.0. Armonk, NY: IBM Corp).

## 3 RESULTS

During the 10‐year period, 170 people with diabetic foot ulcers and clinically suspected osteomyelitis underwent percutaneous bone biopsy. The average subject age was 64.5 years (range 32–93 years). The majority (168, 98.8%) were males. Of these biopsies, 120 (70.6%) were performed on forefoot bones, and the remaining 50 biopsies (29.4%) were performed on hindfoot bones. Table 1 summarizes demographic data.

**TABLE 1:** Summary of demographic data of the included subjects and diagnostic test outcome.

Characteristic	N = 170
Age (years)	64 (32–93)
Gender	
Male	168 (98.8)
Female	2 (1.2)
Bone biopsy site	
Forefoot	120 (70.6)
Hindfoot	50 (29.4)
Bone sample cultured	170 (100)
Positive growth	80 (47.1)
Monomicrobial	43 (53.8)
Polymicrobial	37 (46.3)
Negative growth	90 (52.9)
Bone sample pathology	155 (91.2)
Positive for Osteomyelitis	89 (57.4)
Inadequate sample or equivocal	18 (11.6)
Negative for osteomyelitis	48 (31)
Probe to bone	
Performed	59 (34.7)
Positive	23 (39)
Equivocal	2 (3.4)
Negative	34 (57.6)
Plain radiograph	
Performed	136 (80)
Positive	75 (55.1)
Equivocal	11 (8.1)
Negative	50 (36.8)
Magnetic resonance imaging	
Performed	74 (43.5)
Positive	65 (88)
Equivocal	4 (5.4)
Negative	6 (8.1)

*Note*: Values are presented as median (range) or *n* (%).

Probe to bone tests were reported in 59 persons (34.7%); 23 (39%) probe to bone tests were positive, 2 tests (3.4%) were equivocal, and 34 tests (57.6%) were negative. Foot radiographs were obtained in 136 persons (80%); 75 (55.1%) radiographs were reported as positive for osteomyelitis, 11 (8.1%) were reported as equivocal, and 50 (36.8%) were reported as negative for osteomyelitis. Positive radiographic findings of osteomyelitis include bone destruction and fragmentation and periosteal reaction. MRI of the foot was obtained in 74 persons (43.5%); 65 (88%) MRIs were reported as positive for osteomyelitis, 4 (5.4%) were reported as equivocal, and 6 (8.1%) were reported as negative for osteomyelitis.

All sampled bone (170 specimens, 100%) was sent for culture including aerobic, anaerobic, and fungal cultures. To evaluate for pathological changes of osteomyelitis 155 samples (91.2%) were also sent for histopathological examination. The examination indicated that 89 (57.4%) of pathological bone samples were reported positive for osteomyelitis, 18 samples (11.6%) were reported as either inadequate or equivocal, and 48 (31%) were reported negative for osteomyelitis.

Bone culture was positive in 47.1% of the sampled bone (80 samples), whereas 52.9% (90 samples) did not reveal any microbial growth. In 43 samples (53.8% of the culture‐positive samples), a single microorganism was isolated, and polymicrobial growth was recovered in 37 samples (46.3% of culture‐positive samples).

Out of 89 bone samples with pathological findings consistent with acute osteomyelitis, 51(57.3%) revealed positive microbial growth. There was a strong correlation between pathological findings of osteomyelitis and positive bone culture, Χ^2^ (1, N = 155) = 8.74, *p* = .003. Before sampling, 109 people were on empiric antibiotics, which were withheld in most persons for a variable period. Among persons placed on empiric antibiotics, the choice of antibiotic was modified based on the microbiological results of the sampled bone in 84 (77.1%) persons.

Antibiotics were withheld before the biopsy when it was deemed to be safe at the discretion of the primary team. This was done in an effort to improve biopsy yield and reduce the chance of a culture‐negative biopsy. Based on prior use of antibiotics and withholding period before biopsy, persons were divided into four groups: persons without prior antibiotic treatment (61 persons), persons in whom antibiotics were withheld for >7 days before biopsy (29 persons), persons in whom antibiotics were withheld between 3–7 days before biopsy (31 persons), and persons in whom antibiotics were withheld <3 days before biopsy(49 persons). There was no statistical difference in the microbial yield of the bone culture among these groups of persons, Χ^2^ (3, N = 170) = 0.33, *p* = .954.

Among monomicrobial samples (*n* = 43), Gram‐positive bacteria were isolated from 33 samples (76.7%) with methicillin‐sensitive *Staphylococcus aureus* (MSSA) as the most common pathogen in this group (14 samples, 32.6%) followed by methicillin‐resistant *Staphylococcus aureus* (MRSA), which was isolated from eight samples (18.6%). Gram‐negative bacteria were isolated from eight samples (18.6%) with *Morganellaceae* as the most common pathogen in this group (six samples, 13.9%). *Escherichia coli* was isolated from two samples (4.7%). Anaerobes were isolated from a single sample (2.3%). Table 2 summarizes the bacterial spectrum isolated from positive bone culture samples.

**TABLE 2:** Summary of the microbial spectrum isolated from positive bone culture samples.

	Monomicrobial pathogens (*n* = 43)	Polymicrobial pathogens (*n* = 37)	Positive culture (*n* = 80)
Gram‐positive bacteria			
*Gram‐positive bacteria*	33 (76.7)	24 (64.9)	57 (71.3)
*Staphylococcus* species	28 (65.1)	16 (43.2)	56 (70)
MRSA	8 (18.6)	2 (5.4)	10 (12.5)
MSSA	14 (32.6)	8 (21.6)	22 (27.5)
*S. epidemis*	6 (13.9)	7 (18.9)	13 (16.3)
*Enterococcus species*	2 (4.7)	15 (40.5)	17 (21.3)
*Corynebacterium*	2 (4.7)	12 (32.4)	14 (17.5)
*Streptococcus*	1 (2.3)	14 (37.8)	15 (18.8)
Gram‐negative bacteria			
*Gram‐negative bacteria*	8 (18.6)	24 (64.9)	32 (40)
*Morganellaceae*	6 (13.9)	13 (35.1)	18 (22.5)
*Proteus* species	5 (11.6)	9 (24.3)	14 (17.5)
*Morganella* species	1 (2.3)	3 (8.1)	4 (5)
*Providencia stuartii*	0	1 (2.7%)	1 (1.3)
*E. coli*	2 (4.7)	7 (18.9)	9 (11.3)
*Klebsiella species*	0	5 (13.5)	5 (6.3)
*Pseudomonas* species	0	3 (8.1)	3 (3.8)
*Alcaligenes species*	1 (2.3)	0	1 (1.3)
Anaerobes	1 (2.3)	3 (8.1)	4 (5)
Fungal agents	0	2 (5.4)	2 (2.5)

*Note*: Values are presented as *n* (%). Abbreviations: MRSA, methicillin‐resistant *Staphylococcus aureus*; MSSA, methicillin‐sensitive *Staphylococcus aureus*.

Polymicrobial growth was recovered from 37 samples (46.3% of all culture‐positive samples). Gram‐positive pathogens were isolated from 24 polymicrobial samples (64.9%) with *Enterococcus* species as the most frequently isolated pathogens identified in 15 samples (40.5%) followed by *Streptococcus* species (14 samples, 37.8%). MSSA was isolated from eight samples (21.6%) and MRSA was isolated from two samples (5.4%). Gram‐negative pathogens were equally isolated from polymicrobial samples (24 samples, 64.9%) with *Morganellaceae* species as the most frequently identified Gram‐negative pathogens (13 samples, 35.1%). *E. coli* was isolated from seven samples (18.9%). Three samples revealed anaerobes.

## 4 DISCUSSION

Diabetic foot infection is a common complication of diabetes, affecting up to 34% of persons with diabetes.^11^ Almost half of diabetic foot ulcers develop clinically significant infections, and 20% of persons with infected ulcers develop osteomyelitis, the primary risk factor for amputation in persons with diabetes.^12^, ^13^ Early recognition and effective treatment of osteomyelitis can potentially avoid amputation in affected diabetics. Several diagnostic clinical and radiological tests have been suggested to diagnose osteomyelitis with variable accuracy. Obtaining a bone sample from the potentially affected bone at the time of surgical debridement or percutaneous biopsy remains the gold standard for diagnosis of osteomyelitis. Microbiological examination of the bone specimen provides valuable information regarding the offending pathogens and aids in selection of narrow‐spectrum antibiotics, which may improve clinical outcomes.^14^, ^15^, ^16^ Several studies describe significant discrepancy between soft tissue and bone cultures, emphasizing the role of bone biopsy in management of infected diabetic foot ulcers with suspected osteomyelitis.^17^, ^18^

Fluoroscopy‐guided bone biopsy allows accurate sampling of the suspected affected bone, and when performed through intact skin away from the ulcer site, provides accurate speciation of the responsible pathogens with minimal ulcer flora contamination. However, the microbiologic yield of bone biopsy is variable with a yield range of 56%–93%.^18^, ^19^, ^20^ In our study, bone biopsy microbiology culture yield was slightly lower than what has been reported by previous studies with 47.1% of samples positive for one or more microorganisms. Parks et al reported 26% of positive bone cultures to be monomicrobial.^21^ However, in our study, 53.8% of the culture‐positive samples yielded a single pathogen, whereas 46.3% of culture‐positive samples revealed polymicrobial growth. The percentage of monomicrobial samples in our study was higher than what has been reported in previous studies and may be related to technical factors such as routine avoidance of the ulcer bed by the biopsy needle, therefore minimizing potential ulcer flora contamination.

Gram‐positive bacteria were the most frequently isolated bacteria from positive bone cultures and were present in 57 samples (71.3%). Gram‐positive bacteria were isolated from 76.7% of monomicrobial samples and from 64.9% of polymicrobial samples. *Staphylococcus aureus* was the most frequently isolated pathogen from monomicrobial samples, identified in 51.2% of samples. Approximately one third of samples positive for *Staphylococcus aureus* were classified as methicillin resistant (MRSA). *S. aureus* was isolated from 27% of polymicrobial samples with one fifth classified as methicillin resistant (MRSA). Overall, *S. aureus* was present in 40% of all positive samples, and 12.5% of culture‐positive samples yielded MRSA. These finding are concordant with previous studies, which identify *S. aureus* as the most common pathogen in diabetic foot infection and osteomyelitis in Western countries.^22^, ^23^ Parks et al described similar findings in a Veterans Affairs cohort where *S. aureus* was identified in 49.4% of positive bone cultures with MRSA identified in 17.4% of positive samples.

Gram‐positive and Gram‐negative pathogens were equally isolated from polymicrobial samples, each recovered from 64.9% of the samples. Gram‐positive *Enterococcus* species were the most frequently isolated pathogens in polymicrobial samples identified in 40.5% of these samples followed by Gram‐positive *Streptococcus* species and Gram‐negative *Morganellaceae* with *Proteus* species being the most frequently isolated.

*Pseudomonas* was an infrequent pathogen and was isolated from only three polymicrobial samples, which represent 3.8% of all positive samples. Anaerobes were also infrequent pathogens isolated from a single monomicrobial sample and three polymicrobial samples accounted for 5% of all positive samples.

There are several limitations to this study including its retrospective design. Additionally, the procedure notes did not routinely specify if avoidance of biopsy needle passage through the ulcer was possible, which may have affected the prevalence of polymicrobial infections detected in culture‐positive samples. Finally, 64.1% of persons received antibiotics prior to biopsy. Although antibiotics were withheld in many patients before the procedure, the duration of time between last antibiotic and biopsy was variable. Our analysis showed that the duration of antibiotic withholding did not affect the yield of the bone biopsy. This is consistent with prior studies, which have observed that prebiopsy antibiotic administration had no significant impact on bone biopsy microbial yield.^24^, ^25^, ^26^ This study provides valuable information to treating physicians regarding the various pathogens of diabetic foot osteomyelitis and the frequency of MRSA in these patients. Percutaneous fluoroscopy‐guided biopsy should be routinely considered when there is a strong clinical or imaging suspicion of osteomyelitis in persons with diabetic ulcers including persons treated with empiric antibiotics prior to the procedure.

## 5 CONCLUSION

Percutaneous image‐guided bone biopsy is a low‐risk,^26^ minimally invasive procedure that can provide valuable information about microbial pathogens and therefore enable targeting these pathogens with narrow spectrum antibiotics. *S. aureus* was the most common offending pathogen in our study, similar to previously published literature in the Western Hemisphere.

## AUTHOR CONTRIBUTIONS

All authors have significant contribution to this article as follows: Hassan Al‐Balas: Data analysis and drafting the manuscript. Zeyed A. Metwalli: Review of data analysis and correcting and editing the draft. Aaditya Nagaraj: Data collection. David M. Sada: Data collection and editing the draft.

## CONFLICT OF INTEREST STATEMENT

None of the authors have any relevant financial disclosures or conflict of interest.

## CONSENT FOR PUBLICATION

No consent form was required for this retrospective chart review. No personal information was disclosed.

## Data Availability

The collected data are stored in a secure Veterans Affairs hospital server for a minimum of 5 years and access restricted by federal regulation because of personal identification information.

## References

[1] Blume P , Wu S . Updating the diabetic foot treatment algorithm: recommendations on treatment using advanced medicine and therapies. Wounds. 2018;30(2):29‐35. 29091034

[2] Sanders LJ . Diabetes mellitus. Prevention of amputation. J Am Podiatr Med Assoc. 1994;84(7):322‐328. 8064592 10.7547/87507315-84-7-322

[3] Lipsky BA . Osteomyelitis of the foot in diabetic patients. Clin Infect Dis. 1997;25(6):1318‐1326. 9431370 10.1086/516148

[4] Reiber GE , Pecoraro RE , Koepsell TD . Risk factors for amputation in patients with diabetes mellitus. A Case‐Control Study Ann Intern Med. 1992;117(2):97‐105. 1605439 10.7326/0003-4819-117-2-97

[5] Jang YH , Park S , Park YU , Kwack KS , Jeon SW , Lee HY . Multivariate analyses of MRI findings for predicting osteomyelitis of the foot in diabetic patients. Acta Radiol. 2020;61(9):1205‐1212. doi:10.1177/0284185119897351. 31937109

[6] La Fontaine J , Bhavan K , Lam K , et al. Comparison between Tc‐99m WBC SPECT/CT and MRI for the diagnosis of biopsy‐proven diabetic foot osteomyelitis. Wounds. 2016;28(8):271‐278. 27560470

[7] Lauri C , Tamminga M , Glaudemans A , et al. Detection of osteomyelitis in the diabetic foot by imaging techniques: a systematic review and meta‐analysis comparing MRI, white blood cell scintigraphy, and FDG‐PET. Diabetes Care. 2017;40(8):1111‐1120. 28733376 10.2337/dc17-0532

[8] Alvaro‐Afonso FJ , Lazaro‐Martinez JL , Aragon‐Sanchez J , Garcia‐Morales E , Garcia‐Alvarez Y , Molines‐Barroso RJ . Inter‐observer reproducibility of diagnosis of diabetic foot osteomyelitis based on a combination of probe‐to‐bone test and simple radiography. Diabetes Res Clin Pract. 2014;105(1):e3‐e5. 24857262 10.1016/j.diabres.2014.04.024

[9] Aragon‐Sanchez J , Lipsky BA , Lazaro‐Martinez JL . Diagnosing diabetic foot osteomyelitis: is the combination of probe‐to‐bone test and plain radiography sufficient for high‐risk inpatients? Diabet Med. 2011;28(2):191‐194. 21219428 10.1111/j.1464-5491.2010.03150.x

[10] Lam K , van Asten SA , Nguyen T , La Fontaine J , Lavery LA . Diagnostic accuracy of probe to bone to detect osteomyelitis in the diabetic foot: a systematic review. Clin Infect Dis. 2016;63(7):944‐948. 27369321 10.1093/cid/ciw445

[11] Armstrong DG , Boulton AJM , Bus SA . Diabetic foot ulcers and their recurrence. N Engl J Med. 2017;376(24):2367‐2375. 28614678 10.1056/NEJMra1615439

[12] Lindbloom BJ , James ER , McGarvey WC . Osteomyelitis of the foot and ankle: diagnosis, epidemiology, and treatment. Foot Ankle Clin. 2014;19(3):569‐588. 25129362 10.1016/j.fcl.2014.06.012

[13] Lavery LA , Ryan EC , Ahn J , et al. The infected diabetic foot: Re‐evaluating the Infectious Diseases Society of America diabetic foot infection classification. Clin Infect Dis. 2020;70(8):1573‐1579. 31179491 10.1093/cid/ciz489

[14] Elraiyah T , Tsapas A , Prutsky G , et al. A systematic review and meta‐analysis of adjunctive therapies in diabetic foot ulcers. J Vasc Surg. 2016;63(2 Suppl):46 S‐58 S e1‐2. 10.1016/j.jvs.2015.10.007 26804368

[15] Hingorani A , LaMuraglia GM , Henke P , et al. The management of diabetic foot: a clinical practice guideline by the Society for Vascular Surgery in collaboration with the American Podiatric Medical Association and the Society for Vascular Medicine. J Vasc Surg. 2016;63(2 Suppl):3 S‐21 S. 10.1016/j.jvs.2015.10.003 26804367

[16] Lipsky BA , Berendt AR , Cornia PB , et al. 2012 Infectious Diseases Society of America clinical practice guideline for the diagnosis and treatment of diabetic foot infections. Clin Infect Dis. 2012;54(12):e132‐e173. 22619242 10.1093/cid/cis346

[17] Elamurugan TP , Jagdish S , Kate V , Chandra PS . Role of bone biopsy specimen culture in the management of diabetic foot osteomyelitis. Int J Surg. 2011;9(3):214‐216. 21129507 10.1016/j.ijsu.2010.11.011

[18] Senneville E , Morant H , Descamps D , et al. Needle puncture and transcutaneous bone biopsy cultures are inconsistent in patients with diabetes and suspected osteomyelitis of the foot. Clin Infect Dis. 2009;48(7):888‐893. 19228109 10.1086/597263

[19] Aslangul E , M'Bemba J , Caillat‐Vigneron N , et al. Diagnosing diabetic foot osteomyelitis in patients without signs of soft tissue infection by coupling hybrid 67Ga SPECT/CT with bedside percutaneous bone puncture. Diabetes Care. 2013;36(8):2203‐2210. 23514729 10.2337/dc12-2108 PMC3714532

[20] Couturier A , Chabaud A , Desbiez F , et al. Comparison of microbiological results obtained from per‐wound bone biopsies versus transcutaneous bone biopsies in diabetic foot osteomyelitis: a prospective cohort study. Eur J Clin Microbiol Infect Dis. 2019;38(7):1287‐1291. 30980264 10.1007/s10096-019-03547-6

[21] Parks C , Nguyen S . Bacteriologic analysis of bone biopsy from diabetic foot infections within a VA patient population. Foot (Edinb). 2019;38:1‐3. 30530010 10.1016/j.foot.2018.10.004

[22] Kim JJ , Lydecker A , Dave R , Bork JT , Roghmann MC . Diabetic foot infections: local prevalence of and case‐control study of risk factors for methicillin‐resistant Staphylococcus aureus and Pseudomonas aeruginosa. Open forum. Infect Dis. 2020;7(10):ofaa412. 10.1093/ofid/ofaa412 PMC7588104 33134411

[23] Reveles KR , Duhon BM , Moore RJ , Hand EO , Howell CK . Epidemiology of methicillin‐resistant Staphylococcus aureus diabetic foot infections in a large academic hospital: implications for antimicrobial stewardship. PLoS One. 2016;11(8):e0161658. 27556897 10.1371/journal.pone.0161658 PMC4996514

[24] Zhorne DJ , Altobelli ME , Cruz AT . Impact of antibiotic pretreatment on bone biopsy yield for children with acute hematogenous osteomyelitis. Hosp Pediatr. 2015;5(6):337‐341. 26034165 10.1542/hpeds.2014-0114

[25] Crisologo PA , La Fontaine J , Wukich DK , Kim PJ , Oz OK , Lavery LA . The effect of withholding antibiotics prior to bone biopsy in patients with suspected osteomyelitis: a meta‐analysis of the literature. Wounds. 2019 Aug;31(8):205‐212. 31356178

[26] Wu JS , Gorbachova T , Morrison WB , Haims AH . Imaging‐guided bone biopsy for osteomyelitis: are there factors associated with positive or negative cultures? AJR Am J Roentgenol. 2007;188(6):1529‐1534. 17515372 10.2214/AJR.06.1286

